# Testosterone activates glucose metabolism through AMPK and androgen signaling in cardiomyocyte hypertrophy

**DOI:** 10.1186/s40659-021-00328-4

**Published:** 2021-02-05

**Authors:** Mayarling Francisca Troncoso, Mario Pavez, Carlos Wilson, Daniel Lagos, Javier Duran, Sebastián Ramos, Genaro Barrientos, Patricio Silva, Paola Llanos, Carla Basualto-Alarcón, B. Daan Westenbrink, Sergio Lavandero, Manuel Estrada

**Affiliations:** 1 Programa de Fisiología Y Biofísica, Facultad de Medicina, Instituto de Ciencias Biomédicas (ICBM), Universidad de Chile, 8389100 Independencia, Santiago Chile; 2 Faculty of Health Science, Universidad Central de Chile, Santiago, Chile; 3 Institute for Research in Dental Sciences, Faculty of Dentistry, Universidad de Chile, Santiago, Chile; 4 Departamento de Ciencias de la Salud, Universidad de Aysén, 5951537 Coyhaique, Chile; 5 Departamento de Anatomía y Medicina Legal, Facultad de Medicina, Universidad de Chile, 8389100 Santiago, Chile; 6 Department of Cardiology, University Medical Center Groningen, Groningen, The Netherlands; 7 Advanced Center for Chronic Diseases (ACCDiS), Facultad Ciencias Químicas y Farmacéuticas and Facultad de Medicina, Universidad de Chile, Santiago, Chile; 8 Department of Internal Medicine (Cardiology Division), University of Texas Southwestern Medical Center, Dallas, TX USA

**Keywords:** Testosterone, AMP-activated protein kinase, Glucose transport, Glycolysis, Androgen receptor, Cardiac hypertrophy

## Abstract

**Background:**

Testosterone regulates nutrient and energy balance to maintain protein synthesis and metabolism in cardiomyocytes, but supraphysiological concentrations induce cardiac hypertrophy. Previously, we determined that testosterone increased glucose uptake—via AMP-activated protein kinase (AMPK)—after acute treatment in cardiomyocytes. However, whether elevated glucose uptake is involved in long-term changes of glucose metabolism or is required during cardiomyocyte growth remained unknown. In this study, we hypothesized that glucose uptake and glycolysis increase in testosterone-treated cardiomyocytes through AMPK and androgen receptor (AR).

**Methods:**

Cultured cardiomyocytes were stimulated with 100 nM testosterone for 24 h, and hypertrophy was verified by increased cell size and mRNA levels of β-myosin heavy chain (*β-mhc*). Glucose uptake was assessed by 2-NBDG. Glycolysis and glycolytic capacity were determined by measuring extracellular acidification rate (ECAR).

**Results:**

Testosterone induced cardiomyocyte hypertrophy that was accompanied by increased glucose uptake, glycolysis enhancement and upregulated mRNA expression of hexokinase 2. In addition, testosterone increased AMPK phosphorylation (Thr172), while inhibition of both AMPK and AR blocked glycolysis and cardiomyocyte hypertrophy induced by testosterone. Moreover, testosterone supplementation in adult male rats by 5 weeks induced cardiac hypertrophy and upregulated *β-mhc*, *Hk2* and *Pfk2* mRNA levels.

**Conclusion:**

These results indicate that testosterone stimulates glucose metabolism by activation of AMPK and AR signaling which are critical to induce cardiomyocyte hypertrophy.

**Supplementary Information:**

The online version contains supplementary material available at 10.1186/s40659-021-00328-4.

## Background

Testosterone, the main anabolic steroid hormone, is a key metabolic regulator by controlling nutrient and energy balance to maintain protein synthesis in cardiomyocytes [1–4]. Diverse actions of testosterone on the cardiovascular system are regulated by its circulating levels and by activation of androgen receptor-dependent and -independent mechanisms [5, 6]. Meta-analysis and clinical evidence have indicated an association of anomalous plasma testosterone concentrations with metabolic disorders and cardiovascular diseases [3, 7]. Emergence interventional studies have reported improvements in some cardiometabolic risk factors in patients with low testosterone levels receiving replacement therapy re-establishing their hormone at physiological levels [8, 9]. It has also been shown that high doses of testosterone and its synthetic analogs induces cardiac hypertrophy [10–12]. Despite these clinical evidences, there is limited information on the signaling pathways interlinking metabolism and growth mediated by testosterone in cardiomyocytes.

Testosterone has important physiological effects in the heart as this hormone coordinately regulates metabolic pathways associated with energy requirements during cardiomyocyte growth [13]. Because the heart must maintain a constant functional cardiac output, under chronic anabolic stimulation, cardiomyocytes metabolically adapt energy-encoded components and energetic substrates used to produce ATP [14, 15]. Under normal conditions, energy in the heart is mainly produced through fatty acid β-oxidation and, to a lesser extent, through glucose oxidation [16, 17]. However, in hypertrophy there is an increase in glucose metabolism in cardiomyocytes [18]. Higher glucose incorporation promotes its utilization by modulating ATP-generating pathways and transcriptional programs of gene expression associated with glycolytic machinery [19, 20]. Glycolysis is controlled by several mechanisms mediated by AMP-activated protein kinase (AMPK). The increase in the rate of glycolysis from glucose involves modulation of glucose transport and positive regulation of key glycolytic enzymes [21]. Glycolysis triggers glucose uptake, which in cardiomyocytes is mediated by the glucose transporters GLUT1 and GLUT4 [22]. It has been shown that different pro-hypertrophic stimuli induce GLUT4 translocation and glucose uptake during cardiomyocyte growth [19, 20, 23]. Once glucose is transported into cardiomyocytes, it is phosphorylated by hexokinase 2 (HK2), a glucose rate-limiting step enzyme, and thereby can enter diverse metabolic pathways [19, 23, 24]. Inside the cell, the glucose phosphorylated by HK is metabolized to pyruvate. Glycolysis generates acetyl-CoA to produce NADH and FADH2 via the citric acid cycle, which are later used by mitochondria to generate ATP through the electron transport chain [25].

Testosterone activates the intracellular androgen receptor (AR), a classical action mechanism that requires transcription and translation of new proteins. AR stimulation results in the expression of energy-encoded components of the intracellular energy generation machinery in skeletal muscle [26], prostate cells [27], and cardiac cells [5]. Sato et al*.* (2008) showed in skeletal muscle, that testosterone activates glucose metabolism, increasing the levels of GLUT4 protein and its translocation to the plasma membrane [28]. Furthermore, testosterone promotes the expression of energy-encoded components for ATP generation [26], and modulates the activity of HK and phosphofructokinase (PFK), which are critical glycolytic enzymes [28]. Despite cardiomyocyte hypertrophy induced by several pro-hypertrophic stimuli is commonly associated with a metabolic shift from β-oxidation of fatty acids to glucose consumption [29], the anabolic effects of testosterone involving glucose metabolism in cardiomyocyte hypertrophic growth has been poorly explored.

AMPK, a serine/threonine kinase, is considered the main intracellular energy sensor that regulates cardiomyocyte metabolism [30]. Differential actions of AMPK are associated with upstream metabolic regulators and downstream targets in cardiomyocytes. AMPK promotes glycolysis by increasing GLUT4-mediated glucose uptake [31]. Recently, we have shown that short-term stimulation (30–120 min) of cardiomyocytes with testosterone induces glucose uptake via GLUT4. This process is mediated by the activation of both CaMKII and AMPK [32]. However, whether testosterone increases glucose uptake in hypertrophied-long term stimulation conditions is still unknown.

Testosterone activates cooperative mechanisms interlinking transcription and transduction signaling pathways involving AMPK and peroxisome proliferator-activated receptor-γ coactivator 1α (PGC-1α) to transcriptionally regulate glucose metabolism in prostate cancer [33, 34] and skeletal muscle cells [34]. Cardiac hypertrophy has been widely associated with an increase in glucose utilization and glycolysis [19, 23], a switch in nutrient selection that has traditionally been considered as pathological and maladaptive. Despite this previous evidence some research shows that, rather, an increase in glucose utilization during aging, ischemia or heart failure seems to protect from increased damage [35, 36]. Although testosterone clearly influences cardiomyocyte growth and metabolism, the underlying metabolic signaling involved in long term effects remains unknown.

In this work, we evaluated the role of testosterone on glucose metabolism. Our hypothesis was that glucose uptake and glycolysis increase in testosterone-treated cardiomyocytes through AMPK and AR signaling.

## Materials and methods

### Reagents

Testosterone, 5-bromo-2-deoxyuridine (BrdU), compound C (CC), bicalutamide, indinavir, insulin, 2-NBDG [2-(N-(7-nitrobenz-2-oxa-1, 3-diazol-4-yl) amino)-2-deoxyglucose], Rhodamine/Phalloidin, RIPA and Dako mounting medium were from Thermo-Fisher Scientific (Rockford, IL, USA). Anti-β actin antibody (Monoclonal, mouse anti-rat) and PhosSTOP was purchased from Sigma-Aldrich Chemical Company (St. Louis, MO, USA). HK2 (Monoclonal, mouse anti-rat), anti-phospho-AMPK Thr 172 (Polyclonal, rabbit anti-rat) and anti-total AMPK (Polyclonal, rabbit anti-rat) antibodies were from Cell Signaling Technology (Danvers, MA, USA). The primers used in this study were synthesized and provide from Integrated DNA Technology (Danvers, MA, USA). Paraformaldehyde were from Electron Microscopy Sciences (Hatfield, PA, USA).

### Bioethics statement

Rats were bred in the Animal Breeding Facility of the Faculty of Medicine, University of Chile. All procedures involving neonatal and adult rats used in this study were approved by the Institutional Animal Care and Use Committee (Faculty of Medicine, University of Chile, protocol CBA # 0768 FMUCH, March 2015) in accordance with the National Institutes of Health Guide for the Care and Use of Laboratory Animals.

### Primary culture of neonatal rat cardiomyocytes

The primary cultures of neonatal ventricular cardiomyocytes were prepared from hearts of 1–3-day-old Sprague–Dawley rats as described previously [37]. The protocol produces cultures of cardiomyocytes that are at least 90% pure, which is an established cellular model to study cardiac hypertrophy [38]. To prevent fibroblast proliferation, the growth medium was supplemented with 2.5 μM of BrdU. Cardiomyocytes were cultured in growth medium containing DMEM (cat. no. D5796, Sigma) and M-199 (cat. no. M2520) in relation 4:1 supplemented with 10% FBS and 1% penicillin–streptomycin and after 24 h, the cardiomyocytes were cultured in maintenance medium (DMEM:M-199 without FBS and 1% penicillin–streptomycin). Culture medium is designed to contain all the substrates necessary to sustain basal cardiomyocyte metabolism (FFA and carbohydrates).

### Glucose uptake assay

To evaluate glucose uptake induced by testosterone, cells were plated on 25-mm-diameter glass coverslips in 6-well plates (500,000 cells per coverslip). After 24 h, cardiomyocytes were deprived of serum for at least 24 h prior to the experiments. Then, cells were treated with activators or inhibitors, prior to stimulation with testosterone. Cardiomyocytes were incubated in Krebs medium (NaCl [145 mM], KCl [5 mM], CaCl_2_ [6 mM], MgCl_2_ [1 mM], HEPES Na [25 mM], and NaHCO_3_ [10 mM], pH 7.4) without glucose for 20 min and incubated with 2-NBDG (cat. no. N13195) 300 μM, dissolved in Krebs medium without glucose for 20 min at 37 °C, in the dark. The 2-NBDG fluorescence intensity was determined in single cells (at least 100 cells in five different optical fields per experimental condition were analyzed) under an epifluorescence microscope (Zeiss LED, excitation wavelength λ =  ± 480 nm), applying a circular region of interest of 30 × 30 pixels.

### Measurements of extracellular acidification rate

As an estimation of glycolysis, extracellular acidification rate (ECAR) was measured using an Extracellular Flux Analyzer (Seahorse Bioscience XF96 analyzer North Billerica, MA). Briefly, 80,000 cells/well previously treated with vehicle (0.01% ethanol), testosterone (100 nM), bicalutamide (2 μM, 30 min), or CC (2 μM, 30 min) were cultured in 96-well culture plates using the XF GlycoStress® protocol. For glycolytic flux assay, the base medium was supplemented with 2 mM l-glutamine. Glutamine is used to achieve the maximal glycolysis rate. Glucose 10 mM was added to initiate glycolysis and measure glycolytic capacity in the cells. Triplicate measurements were made for each treatment condition. Cell viability, determined after the assays, was nearly indistinguishable among the different conditions, regardless of the presence or absence of exogenous substrates or metabolic inhibitors in the assay medium. Data were obtained using Wave® software (https://www.agilent.com/en/products/cell-analysis/cell-analysis-software/data-analysis/wave-desktop-2-6) and glycolysis and glycolytic capacity were determined by measuring ECAR, which is attributable to the production of lactate by the metabolism of glucose through glycolysis in cells in a basal state compared with cells stimulated with testosterone [39].

### Western blot analysis

Lysates were prepared from cardiomyocytes that were plated on 35-mm plates and serum-starved for 24 h before exposure to testosterone for the indicated times. Cell lysates were prepared with a lysis buffer (RIPA cat. no. 89901, Thermo Scientific and PhosSTOP (cat. no. 4906845001, Sigma) and centrifuging them at 15,000×*g* for 10 min at 4 °C, supernatants were removed and store for quantification of proteins. Protein concentrations were determined by Coomassie Plus kit (cat. no. 23238, Thermo Scientific, Rockford, IL) according to the manufacturer’s instructions. Cell lysate were resolved by SDS-PAGE and were transferred to nitrocellulose Hybond membranes (Amersham Biosciences Corporation, Piscataway, NJ) with a Pierce G2 Fast Blotter (Thermo-Fisher Scientific). After blocking the membranes in 5% non-fat dry milk or 5% BSA, the membranes were incubated overnight at 4 °C with specific primary antibodies against phospho-AMPK Thr 172 (58,5 μg/ml; cat. no. 2531, Cell Signaling), total AMPK (44 μg/ml; cat. no. 2532, Cell Signaling), or HK2 (23,5 μg/ml; cat. no. 2867, Cell Signaling). Next, the membranes were washed and incubated with HRP-conjugated secondary antibody (95–750 μg/ml; anti-rabbit, cat. no. A6154, Sigma) for 1–2 h at room temperature. The protein bands were visualized using the SuperSignal West Pico Chemiluminescent Substrate (Thermo-Fisher Scientific, Rockford, IL, USA) and Westar Supernova (Cyabagen, Bologna, Italy) in the ChemiDoc Imaging System (Bio Rad). Band intensities were determined by densitometry with ImageJ software (NIH, Bethesda, MD, USA). In addition, for protein loading control, the membranes were stripped with Restore PLUS Western Blot Stripping Buffer (Thermo) for 20 min at room temperature and then incubated with primary antibody for β-actin (312–750 μg/ml; cat. no. A5441; Sigma, St. Louis, MO, USA) and performed the incubation with HRP-conjugated secondary antibody (1.875–3.75 mg/ml; anti-mouse, cat. no. A9044, Sigma) and the same protocol described previously for visualization and quantification.

### RNA extraction and RT-qPCR

Total RNA was extracted from cultured cardiomyocytes or heart tissue using 1 mL of TRIZOL reagent (Invitrogen) following the manufacturer’s instructions. RNA purity (absorbance ratio 260 nm/280 nm) and its concentration were determined using a NanoDrop (Thermo-Fisher Scientific, Rockford, IL). cDNA was synthesized from purified mRNA derived from cultured cardiomyocytes by reverse transcription using 1 μg of RNA and SuperScript II (cat. no. 18064022, Invitrogen), following the manufacturer's instructions. qPCRs were run on the StepOne Plus Real Time PCR system (Applied Biosystems) using Power SYBR Green PCR Master Mix (cat. no. 4367659, Applied Biosystems). Serial dilutions of a standard sample were included for each gene to generate a standard curve and after RT-q-PCR for each sample using the cycle protocol: 95 °C for 10 min, 40 cycle (95 °C for 10 s, 58 °C for 20 s, 60 °C for 40 s) and for the melting curve: 95 °C for 15 s, 60 °C for 1 min, 95 °C for 15 s. Primers were design using the BLAST platform. As an amplification control, the *18S* ribosomal RNA (sense primer, 5′-CGACGACCCATTCGAACGTCT-3′; antisense primer, 5′-GCTATTGGAGCATGGAATTACCG-3′) was used. The specific primers for each gene were as follows: *Hk2*: (sense 5′-CCTATGCACTAGCCAACTTC-3′; antisense 5′-CACCGCCGTCACCATAGC-3′), *Pfk2*: (sense 5′-CCTATGCACTAGCCAACTTC-3′; antisense 5′-CACCCGCATCAATCTCATTC-3′). *β-mhc* (sense 5′-AAGTCCTCCCTCAAGCTCCTAAGT-3′; antisense 5′-TTGCTTTGCCTTTGCCC-3′). Data were analyzed using StepOnePlus™ Software v2.3. Relative gene expression was calculated using the 2^–ΔΔCT^ method [40].

### Measurement of cell size

Cells were cultured on gelatin-coated coverslips for 24 h, and then treated for 24 h with testosterone (100 nM), bicalutamide (2 µM) or CC (2 µM). The cardiomyocytes were fixed with 4% paraformaldehyde in phosphate buffer (PBS) for 15 min at room temperature. Then, the cells were washed in PBS 1 × and incubated with Rhodamine Phalloidin (1:300, cat. no. R415, Invitrogen) for 20 min at room temperature. Images were acquired using a Zeiss-Colibri epifluorescence microscope (Zeiss, Germany) and analyzed using ImageJ to determine the cellular area. The fields were selected randomly and blinded in each experimental condition and 5–8 cell were selected of five fields for determination of cellular area. For the measurements, we used at least five different fields from three independent cultures in each condition.

### Transfections

siRNA-AMPKα2 (sc-155985, Sta. Cruz Biotechnology) was used to decrease AMPK protein levels. Transfections were performed using Lipofectamine RNAiMAX (cat. no. 13778150 Invitrogen, Carlsbad, CA, USA) and Opti-MEM Reduced Serum Medium, GlutaMAX Supplement (cat. no. 51985034, Thermo-Fisher Scientific, Rockford, IL, USA), according to the manufacturer’s specifications. The effectiveness of the siRNA for AMPK was verified by immunodetection using specific antibodies to assay the total protein level.

### Cardiac hypertrophy in vivo

Cardiac hypertrophy induced by testosterone in adult rats was performed as described previously [41]. A total of 12 male 8-week-old Sprague–Dawley rats were used, and orchiectomy (ORX) was performed in 8 rats to reduce circulating levels of testosterone. Briefly, to perform the surgery the rats were anesthetized with a mixture of ketamine/xylazine (80 mg·kg^−1^ / 10 mg·kg^−1^, ip), and after that they were allowed to recover for 7 days. Rats were kept at constant temperature under a regular light and dark cycle with free access to food and water. Next, the animals were randomly assigned in three groups: 1) Control, 2) ORX treated with vehicle; and 3) ORX treated with testosterone (125 mg·kg^−1^·week^−1^) for 5 weeks (ORX + T). After treatment, control, ORX and ORX + T rats were euthanized by administering an overdose of sodium pentobarbital (200 mg·kg^−1^). The hearts were dissected and weighed to determine hypertrophy parameters and extract the RNA. Testosterone concentrations were determined by ELISA (cat. no. 582701, Cayman Chemical, Ann Arbor, MI, USA).

### Statistical analysis

Data are expressed as mean ± SEM or as representative experiments performed at least three times independently. Means were compared by repeated-measures analysis of variance (ANOVA), or in the case of two groups, with the Student *t*-test. Multiple means were compared by one-way ANOVA followed by Tukey’s post-hoc analysis. All statistical analyses were conducted using GraphPad Prism 6 software (GraphPad Software Inc., San Diego, CA, USA). A *P* < 0.05 were considered to indicate significative difference.

## Results

### Testosterone induces glucose uptake and glycolysis in hypertrophied cardiomyocytes.

**Fig. 1:**
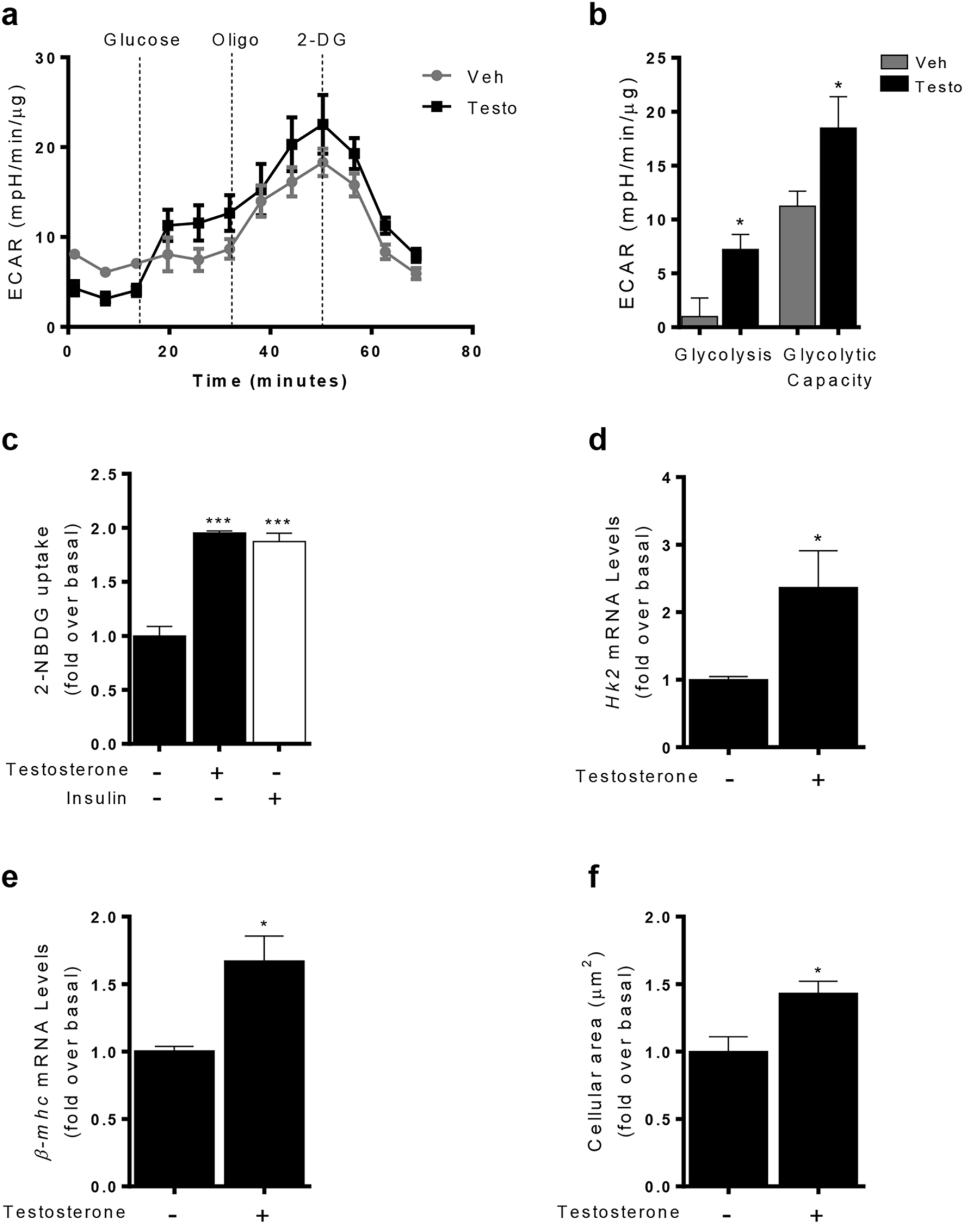
Testosterone promotes glycolysis and glucose uptake in cardiomyocyte hypertrophy. **a** Extracellular acidification rate (ECAR) in cells treated with 100 nM testosterone for 24 h. Traces correspond to ECAR after serial addition of 10 mM glucose, 2 μg·ml^−1^ oligomycin, or 1 μM 2-deoxy-d-glucose (2-DG) respect to time experiment. **b** Glycolysis and maximal glycolytic capacity parameters induced by testosterone (100 nM) after 24 h of stimulation. Triplicate measurements of ECAR were made for each treatment condition. **c** Glucose uptake was measured as 2-NBDG uptake (300 µM) and was normalized to the basal level after exposure to testosterone (100 nM) for 24 h or insulin (100 nM) for 20 min. Cardiomyocytes were stimulated with 100 nM testosterone for 10 h and **d** *Hk2* and **e** *β-MHC* mRNA was determined by RT-qPCR. The mRNA levels were normalized to *18S* mRNA levels and the values shown here correspond with target-gene/*18S* mRNA ratios. **f** Cell size was assessed using the fluorescent dye Rhodamine/Phalloidin. Data are expressed as mean ± SEM of at least three independent experiments. P-values were determined using t-test, except for Fig. 1C that have ANOVA followed by Tukey’s post hoc test; *P < 0.05, ***P < 0.001 vs. control non stimulated cells

**Fig. 2:**
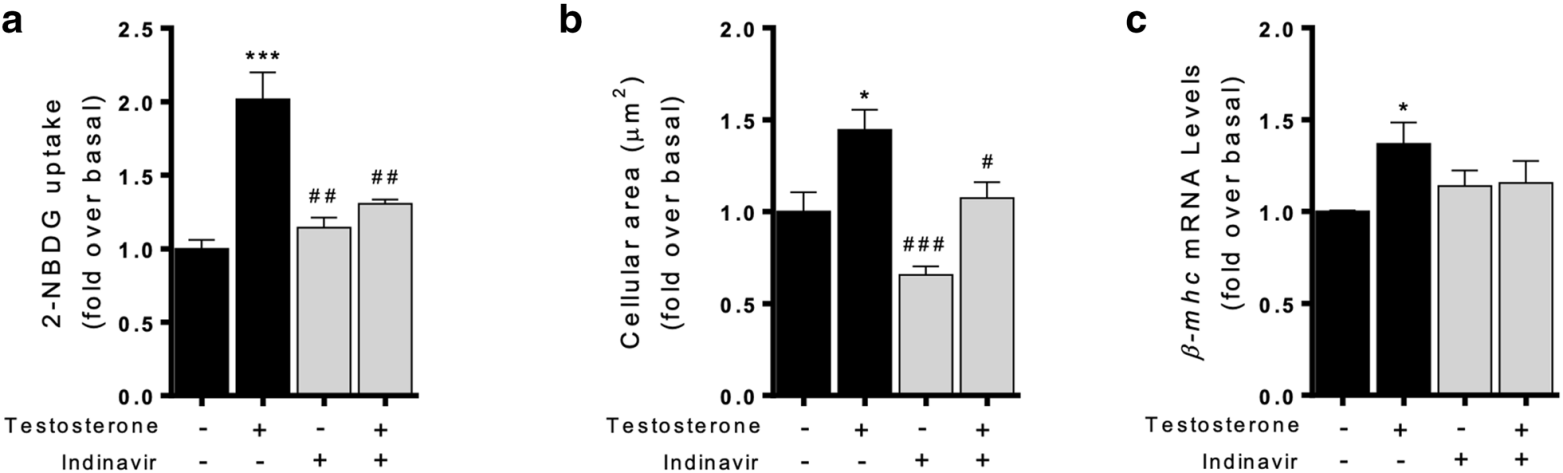
Increased glucose uptake through GLUT4 is required to induce cardiomyocyte hypertrophy by testosterone. **a** Glucose uptake was measured as 2-NBDG uptake (300 µM) and was normalized to the basal level after exposure to 100 nM testosterone for 24 h. Testosterone-induced 2-NBDG uptake was measured in the presence or absence of indinavir. Cardiomyocytes were pretreated with 100 µM indinavir and stimulated with 100 nM testosterone by 24 h. **b** Cellular area was assessed using the fluorescent dye Rhodamine/Phalloidin, and > 100 cells were analyzed in each condition. **c** Cardiomyocytes were stimulated with 100 nM testosterone for 10 h and *β-mhc* mRNA level was determined by RT-qPCR. The mRNA levels were normalized to *18S* mRNA levels (n = 6). Data are expressed as mean ± SEM of at least three independent experiments. *P < 0.05, ***P < 0.001 vs. control; #P < 0.05, ##P < 0.01, ###P < 0.001 vs. testosterone

To determine changes in glucose metabolism upon long-term (24 h) testosterone exposure in cardiomyocytes, we examined glucose uptake and glycolysis. First, glycolysis was assessed by measuring the extracellular acidification rate (ECAR). In this assay, 1 h before the experiments, cells are cultured without glucose or pyruvate and glycolysis is measured after the injection of a saturating dose of glucose (10 mM), and then adjusted so that the non-glycolytic acidification represents only ECAR from glycolysis. The glycolytic capacity is a measure of the cellular ability to maintain its demand for ATP solely through glycolysis, a condition that is achieved by blocking ATP synthase with oligomycin [39]. As expected, testosterone treatment increased both glycolysis and maximal glycolytic capacity compared to control cardiomyocytes (Fig. 1a, b). Next, we evaluate glucose uptake changes upon long-term testosterone exposure (24 h), in cardiomyocytes incubated with the fluorescent glucose analog 2-NBDG. After 24 h of testosterone incubation there is an increased glucose uptake relative to control non-stimulated cardiomyocytes (Fig. 1c). Insulin (100 nM for 30 min) served as positive control of glucose uptake (Fig. 1c). These responses were abolished by preincubation with 2 µM indinavir, a GLUT4 inhibitor (Fig. 2a). To investigate whether the increased intracellular glucose resulting from the augmented uptake rate is metabolized via glycolysis, we analyzed, by RT-qPCR the gene expression of *Hk2* a key enzyme regulating glycolysis [24]. Our results showed that testosterone significantly increased mRNA levels of *Hk2* (Fig. 1d)*.* In order to confirm further the hypertrophic effects of testosterone, we evaluated well-characterized indicators of cardiomyocyte hypertrophy including cell size and mRNA levels of β-myosin heavy chain (*β-mhc)* [38]. Testosterone treatment by 10 h increases the mRNA expression levels of *β-mhc* (Fig. 1e). Furthermore, stimulation with 100 nM testosterone for 24 h resulted in significant increases in cardiomyocyte size reaching 1,fivefold compared to control non stimulated cells (Fig. 1f). The role of GLUT4-mediated glucose uptake in regulating cardiomyocyte growth induced by testosterone was next assessed by using indinavir. The increase in hypertrophic markers such as cellular area and *β-mhc mRNA levels* were prevented by treating cells with 2 µM indinavir prior testosterone stimulation for 24 h (Fig. 2b, c).

### Androgen receptor activation is required for the long-term effects of testosterone on glucose metabolism in cardiomyocytes

**Fig. 3:**
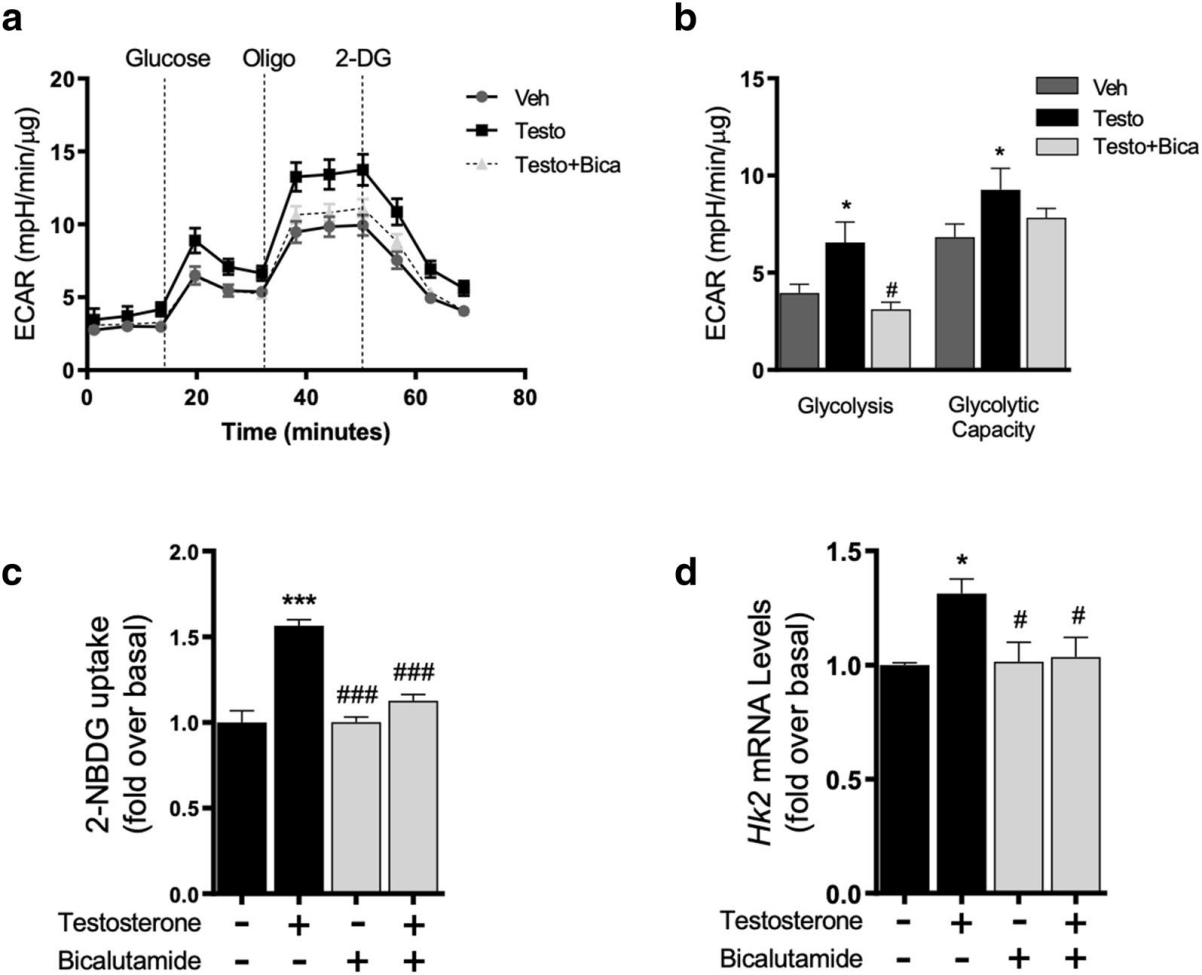
AR is required for glucose metabolic changes in testosterone-induced cardiomyocyte hypertrophy. **a** ECAR kinetics in cells treated with bicalutamide (2 µM) and testosterone 100 nM for 24 h. **b** The increase in glycolysis and maximal glycolytic capacity induced by 100 nM testosterone were blocked by bicalutamide. **c** Cardiomyocytes were stimulated with 100 nM testosterone for 24 h and were then incubated with 2-NBDG for 20 min, intracellular fluorescence was measured in the presence or absence of bicalutamide (2 µM). AR inhibition abolished testosterone-stimulated glucose uptake. **d** Cardiomyocytes were pretreated for 30 min with bicalutamide (2 µM). Levels of mRNA for *Hk2* were evaluated in cells treated with testosterone 100 nM for 10 h in the presence or absence of bicalutamide (2 µM). Data are expressed as mean ± SEM of at least three independent experiments. P-values were determined using ANOVA followed by Tukey’s post hoc test; *P < 0.05, ***P < 0.001 vs. control; #P < 0.05, ###P < 0.001 vs. testosterone treated cells

To assess whether increased glucose metabolism in cardiomyocytes depends on AR activation, cells were pretreated with the AR inhibitor bicalutamide (2 µM, 30 min) prior to testosterone stimulation. As shown in Fig. 3a and b, AR inhibition with bicalutamide abolished the effects testosterone on glycolysis. The glycolytic capacity is increased by testosterone, but the effect is not present in testosterone with bicalutamide*.* In concordance with these results, glucose uptake induced by testosterone stimulation for 24 h was inhibited in cardiomyocytes pretreated with bicalutamide (Fig. 3c). Additionally, we determined the *Hk2* mRNA levels after 10 h of testosterone incubation and bicalutamide abolished testosterone-induced increases in mRNA levels of *Hk2* (Fig. 3d)*.* These results suggest that AR activation signaling is required for increasing glucose uptake and glucose metabolism triggered by testosterone.

### AMPK regulates glycolysis and glucose uptake activated by testosterone in cardiomyocytes

**Fig. 4:**
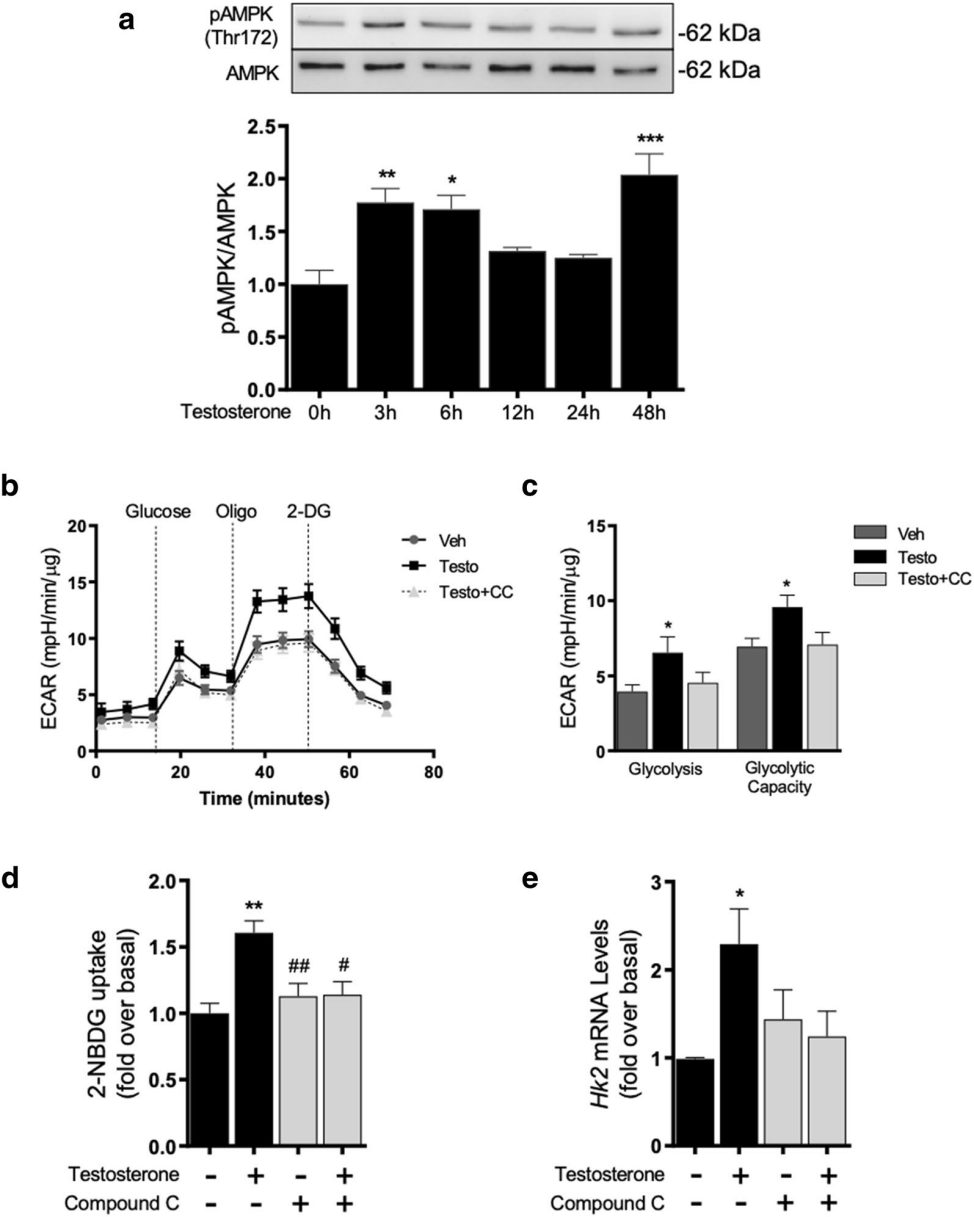
Testosterone-induced glucose metabolism is mediated by AMPK. **a** Western blot showing the phosphorylation levels of AMPK at residue Thr172 in response to 100 nM testosterone stimulation for 0, 3, 6, 12, 24 and 48 h. Ratio of p-AMPK to total AMPK protein as determined by densitometry. **b** ECAR was measured using a Seahorse XF analyzer and values were normalized using the total protein per well. ECAR kinetics in cells treated with 2 µM CC and 100 nM testosterone for 24 h. **c** Glycolysis and maximal glycolytic capacity parameters increased by testosterone were blocked by pretreatment with CC. **d** Cardiomyocytes were stimulated with 100 nM testosterone for 24 h and were then incubated with 2-NBDG for 20 min. Intracellular fluorescence was measured in the presence or absence of CC (2 µM). AMPK inhibition blocked the increase in glucose uptake induced by testosterone. **e** Cells were incubated for 30 min with 2 µM CC and then treated with 100 nM testosterone for 10 h. mRNA levels of *Hk2* were assessed by RT-qPCR and normalized to *18S* mRNA. Data are expressed as mean ± SEM of at least three independent experiments. P-values were determined using ANOVA followed by Tukey’s post hoc test; *P < 0.05, **P < 0.01, ***P < 0.001 vs. control; #P < 0.05, ##P < 0.01 vs. testosterone treated cells

In cardiomyocytes AMPK is a central intracellular energy sensor that regulates metabolic pathways associated with glucose metabolism and glycolysis [30]. To determine whether testosterone activates AMPK, we assessed the kinetics of AMPK phosphorylation at residue Thr172, which is essential for its kinase activity [42, 43]. As shown in Fig. 3a, testosterone (100 nM) increased AMPK phosphorylation, peaking at 3 h to 6 h*,* and returning to basal levels after 12 h of hormone stimulation. Moreover, we observed that AMPK phosphorylation was significantly increased in twofold after 48 h of testosterone stimulation as compared to control non stimulated cells (Fig. 4a). To determine the role of AMPK in testosterone-stimulated glucose metabolism in long term testosterone treatments, cells were pretreated with 2 µM CC, a pharmacological AMPK inhibitor [44]. Glycolysis and maximal glycolytic capacity increased by testosterone were decreased by inhibition of AMPK (Fig. 4b, c). Testosterone (100 nM) stimulation for 24 h increased glucose uptake and these responses were significantly suppressed by AMPK inhibition (Fig. 4d). Moreover, treatment of cardiomyocytes with 100 nM testosterone for 10 h increased the mRNA levels of *Hk*2, which was inhibited by pretreating cells with 2 µM CC prior testosterone stimulation (Fig. 4a). These results suggest that in cardiomyocytes AMPK activation is involved in glucose metabolism triggered by testosterone treatment.

### Increased glucose uptake and AMPK are required in testosterone-induced cardiomyocyte hypertrophy

**Fig. 5:**
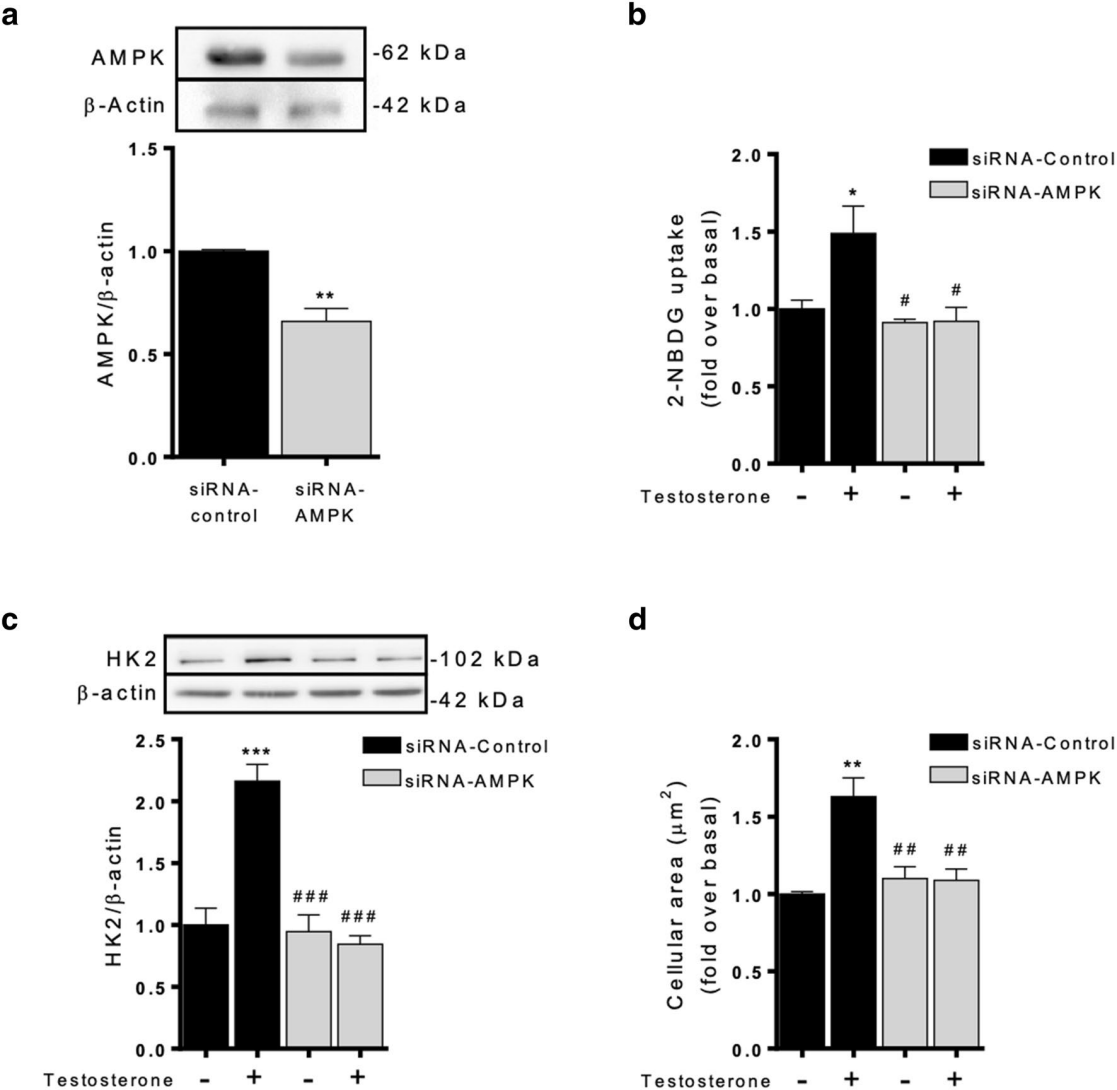
AMPK is required for testosterone-mediated glucose metabolism and hypertrophy. **a** Cardiomyocytes were transfected with siRNA-control or siRNA-AMPKα2 (20 nM) and protein levels were determined by Western blot. **b** Transfected cardiomyocytes were stimulated with 100 nM testosterone for 24 h and were then incubated with 2-NBDG for 20 min prior to glucose uptake measurements. **c** Cells were transfected and stimulated with testosterone 100 nM for 24 h and were evaluated the levels of HK2 protein by Western Blot. **d** Transfected cardiomyocytes were stimulated with 100 nM testosterone by 24 h and cellular area was measured using the fluorescent dye Rhodamine/Phalloidin and > 100 cells were analyzed in each condition. Data are expressed as mean ± SEM of at least three independent experiments. P-values were determined using t-test for Fig. 4a and ANOVA followed by Tukey’s post hoc test for the rest; *P < 0.05, **P < 0.01, ***P < 0.001 *vs*. control; #P < 0.05, ##P < 0.01, ###P < 0.001 *vs*. testosterone

**Fig. 6:**
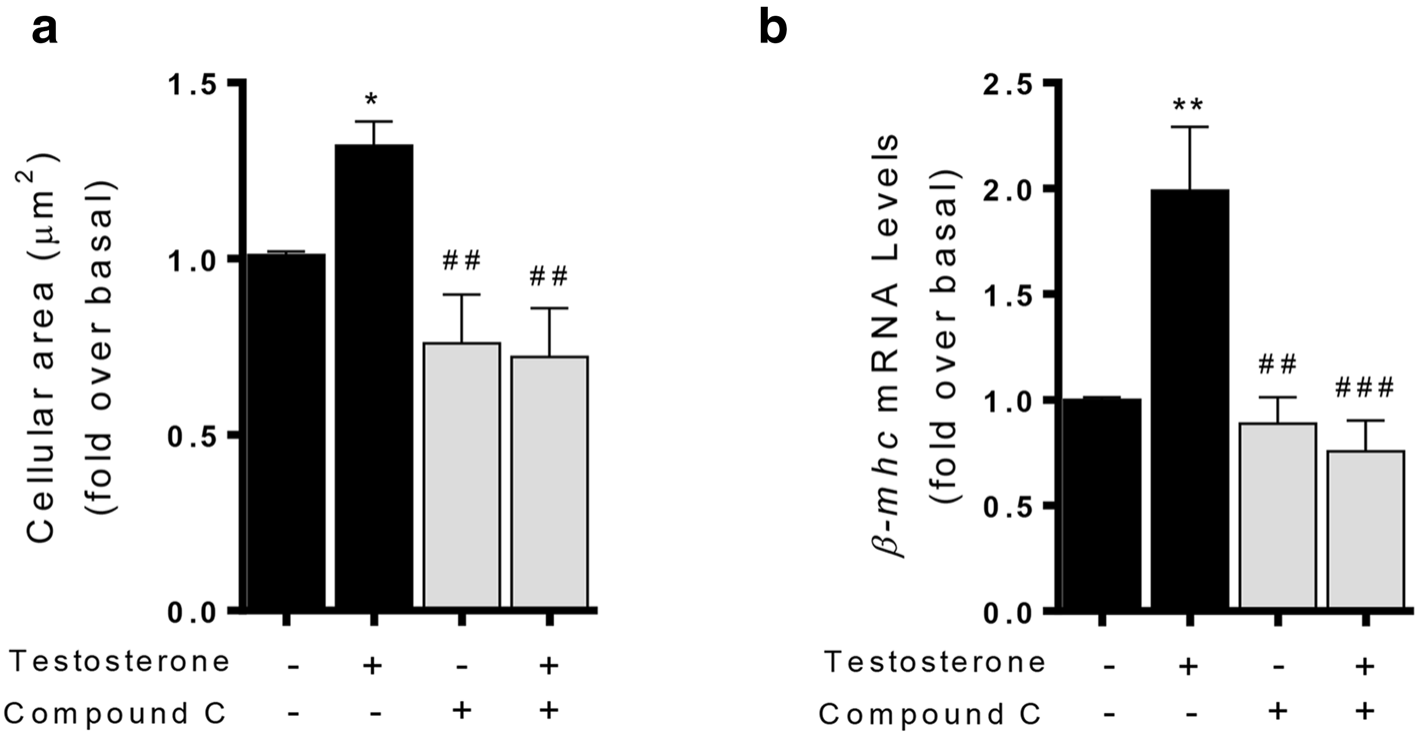
AMPK is required in testosterone-induced cardiomyocyte hypertrophy. Cardiomyocytes were pretreated with 2 µM CC and stimulated with 100 nM testosterone by 24 h. **a** Cardiomyocyte cellular area. **b** *β-mhc* mRNA levels were evaluated in cells treated with 100 nM testosterone for 10 h in the presence or absence of CC (2 µM). The mRNA levels were normalized to 18S mRNA expression. Data are expressed as mean ± SEM of at least three independent experiments. *P < 0.05, **P < 0.01 vs. control; ##*P* < 0.01, ###*P* < 0.001 vs. testosterone

Next, we investigated whether AMPK activation by testosterone links glucose metabolism and cardiomyocyte hypertrophy. Cardiomyocytes were transfected with siRNA-control or siRNA-AMPKα2 (20 nM) and protein levels were determined by Western blot. Total AMPK levels decreased in cells treated with siRNA-AMPKα2 (Fig. 5a). Transfected cardiomyocytes were stimulated with 100 nM testosterone for 24 h and were afterwards incubated with 2-NBDG for 20 min prior to glucose uptake measurements. AMPK downregulation mediated by siRNA-AMPK abolished the increase in glucose uptake (Fig. 5b), protein levels of HK2 (Fig. 5c) and cardiomyocyte size (Fig. 5d) in response to testosterone treatment. Moreover, in cardiomyocytes the preincubation with 2 µM CC abolished the increase in glucose uptake (Fig. 6a) and *β-mhc* mRNA levels (Fig. 6b) induced by testosterone. Together, these results suggest that cardiomyocyte hypertrophy triggered by testosterone involves increments in glucose uptake and metabolism through AMPK–AR pathways.

### Testosterone increases expression levels of glycolytic enzymes in cardiac hypertrophy in vivo

**Fig. 7:**
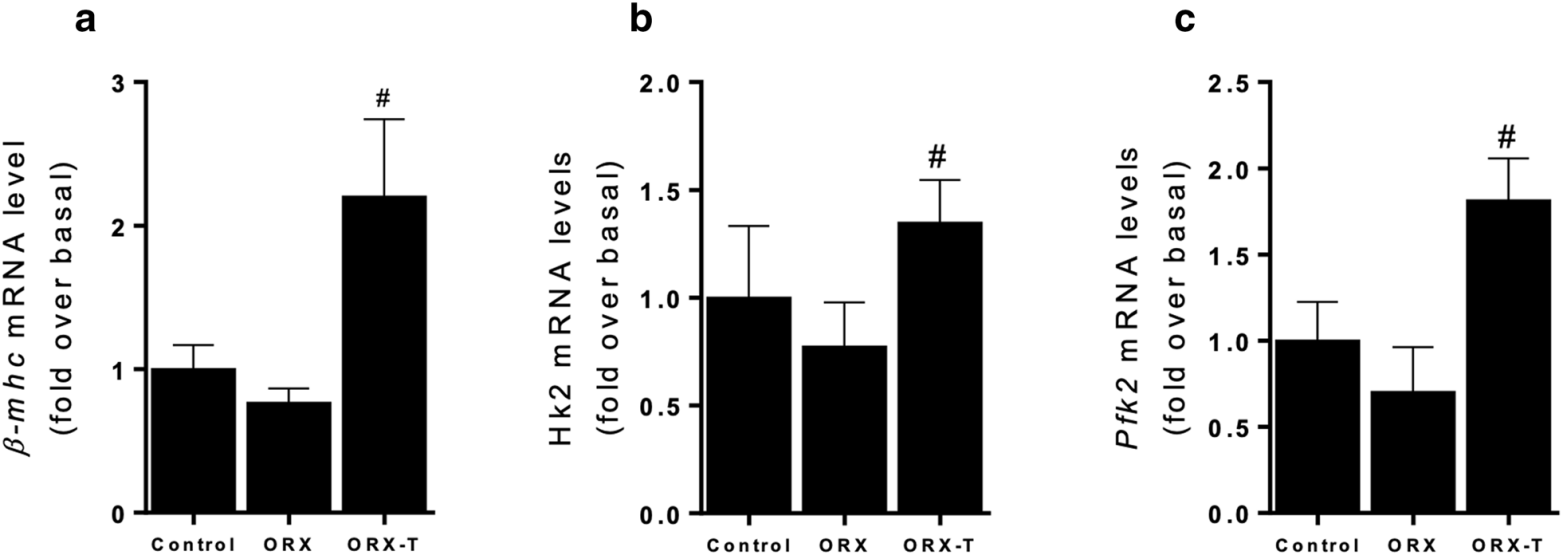
Testosterone increases expression levels of glycolytic enzymes in cardiac hypertrophy in vivo. The rats were ORX and treated with vehicle (ORX) or Testosterone (ORX + T). Control group were performed with normal rats. mRNA levels were assessed using RT-qPCR for **a** β-mhc; **b** *Hk2*; and **c** *Pfk2* (n = 4 rats per each experimental condition). These genes were normalized relative to *18S* mRNA expression, and the values shown here correspond to target-gene/18S mRNA ratios. Data are presented as means ± SEM; P-values were determined using ANOVA followed by Tukey’s post hoc test; #P < 0.05 vs. ORX group

Lastly, we conducted in vivo studies to investigate the physiological relevance of our in vitro findings obtained using cultured cardiac myocytes: we examined mRNA levels of *β-mhc* and glycolytic enzymes *Hk2* and *Pfk2* in control and ORX rats that were or were not administered testosterone. Control rats exhibited plasma testosterone concentrations of 6.23 ± 4.55 ng·ml^−1^, which were significantly higher in the testosterone-treated ORX rats (42.3 ± 17.21 ng·ml^−1^) than in untreated ORX rats (0.13 ± 0.01 ng·ml^−1^). Furthermore, we evaluated the changes in the heart size induced by administration of testosterone in adult rats. In ORX + T rats, the heart weight/body weight ratio increased as compared with ORX and control non treated rats (Additional file 1: Fig. S1). These results indicate that testosterone produced its expected anabolic effects in this hypertrophy model in vivo. Moreover, testosterone supplementation upregulated *β-mhc*, *Hk2* and *Pfk2* mRNA expression levels compared with ORX or control rats (Fig. 7). These results provide insight into actions of testosterone on glucose metabolism in cardiac hypertrophy in vivo.

## Discussion

In this study, we determined that testosterone promotes a change in nutrient use, by increasing glucose metabolism through AMPK and AR signaling during cardiomyocyte hypertrophy. These findings suggest that, during hypertrophy, testosterone increases glucose consumption, which could be a necessary step for energy production and consequent cellular growth in cardiomyocytes.

**Fig. 8:**
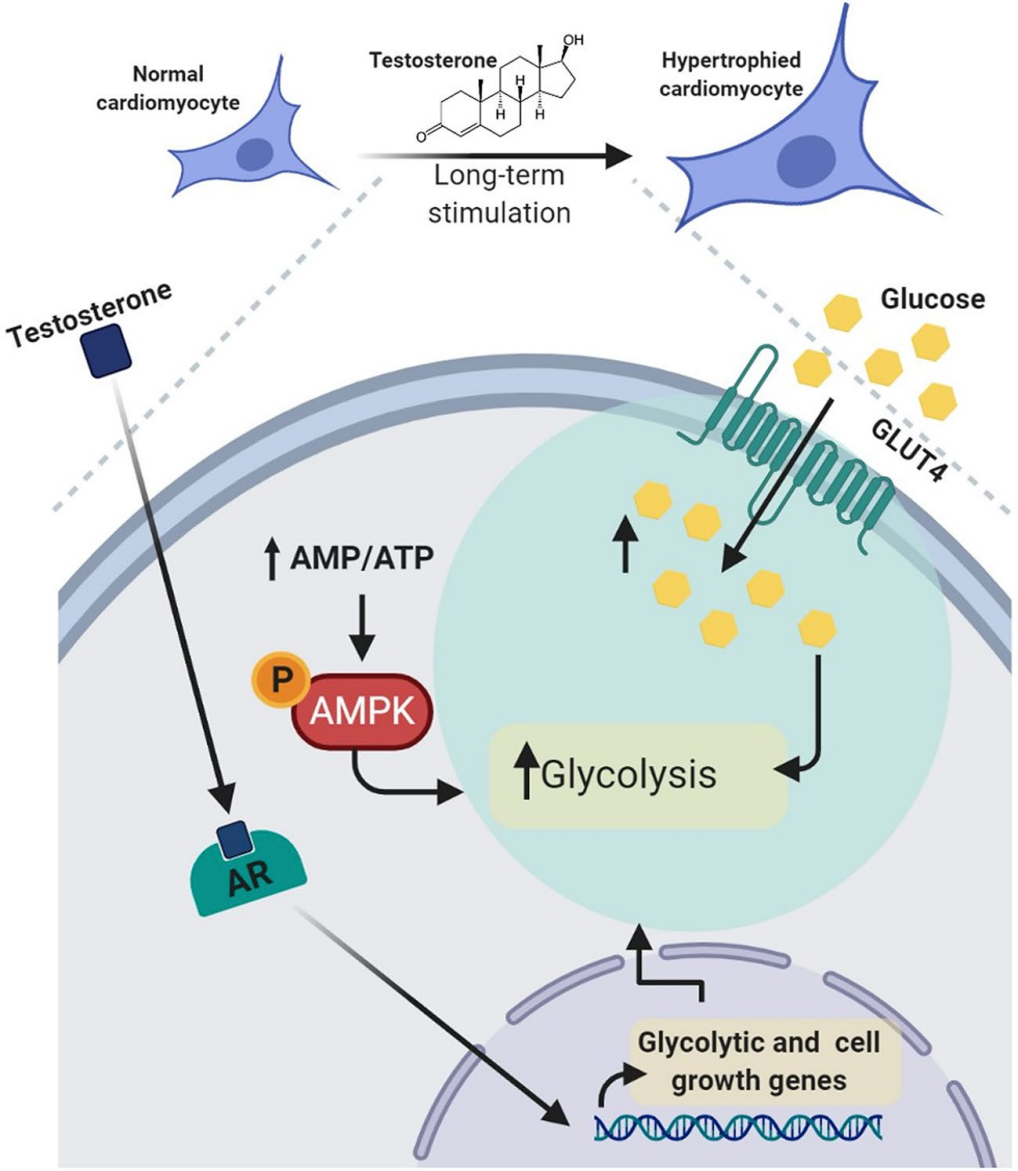
Proposed mechanism for testosterone action on glucose metabolism and cell growth during cardiomyocyte hypertrophy. Testosterone increases glucose uptake and glycolysis through the energy sensor AMPK and the androgen receptor signaling to deliver metabolic information toward transcription factors that coordinately regulate metabolism and gene expression during cardiomyocyte hypertrophy

Regulation of cell metabolism is a main function of testosterone in the heart, and it does so by continuously controlling the nutrient and energy balance to maintain protein synthesis in cardiomyocytes [45–47]. Previously, we have shown that, in cardiomyocytes, a short-term testosterone stimulation (30 min) quickly increases glucose uptake, which resulted from AMPK and CaMKII activation [32]. However, the metabolic outcome or the potential changes in glucose metabolic machinery in cardiomyocyte hypertrophy induced by long term testosterone stimulation had not been studied. In this study, we determined that testosterone promotes glycolysis and glycolytic gene expression through activation of AMPK and AR. Indeed, our previous study suggested that the ability to modulate glucose uptake in the short term through GLUT4 and AMPK may be an early anabolic signal of testosterone to support the subsequent cell growth. Concordant with this idea, the current study indicates that once hypertrophy is consolidated, the increase in glucose uptake persists and glycolysis is activated, which involves AMPK and AR signaling in cardiomyocytes. As depicted in the proposed model for the mechanism of testosterone action on glucose metabolism and cell growth during cardiomyocyte hypertrophy (Fig. 8).

In cardiomyocytes, the biological actions of testosterone are regulated through activation of AR-dependent and -independent mechanisms [6, 48]. Sedentary lifestyle and over-nutrition to be negatively associated with plasma testosterone levels [3, 49]. In addition, the gain in body fat mass associated with being overweight and obese also increases enzymes such as aromatase and aldo–keto reductase 1C, which metabolize testosterone into estrogens and 5-dihydrotestosterone metabolites that reduce insulin sensitivity [50, 51]. Low testosterone concentrations are correlated with an increased fat mass (particularly central adiposity), reduced insulin sensitivity, impaired glucose tolerance, elevated triglycerides and cholesterol and low HDL-cholesterol, contributing to cardiovascular risk [49, 52]. On the other hand, exogenous administration of high concentrations of androgens also increases cardiovascular risk by inducing cardiac hypertrophy [12, 53].

Our findings suggest that cardiomyocytes display an increased reliance on glycolysis as a source of energy following hypertrophy-inducing testosterone stimulation. Previously, we have shown that short-term (30 min) stimulation of cardiomyocytes with testosterone quickly increases glucose uptake, which results from AMPK and CaMKII activation [32]. Here, we showed that glucose uptake remains elevated during prolonged testosterone stimulation (100 nM for 24 h), which is in agreement with the development of cardiomyocyte hypertrophy [54]. Accordingly, testosterone treated cardiomyocytes show a marked increase in glycolysis. Despite the lower efficacy of glucose oxidation for energy generation, cardiomyocytes require increased nutrient availability to ensure high-level metabolic supply for cell growth and it is obtained through a positive regulation of glucose transporters and glycolytic enzyme activity [23, 55]. Here, our results showed that testosterone upregulates the transcription of the metabolic enzymes HK2 and PFK2, which are involved in glucose metabolism and glucose oxidative capacity in cardiomyocytes [24, 56, 57]. Moreover, we confirmed that GLUT4 is the transporter that mediates this glucose uptake by using indinavir, a GLUT4-specific inhibitor [58].

In agreement with our results, limiting glucose availability, through inhibition of GLUT4-mediated glucose uptake by indinavir, prevented hypertrophic growth in response to testosterone **(**Fig. 2**)**. To explore the potential mechanism involved in the enhanced exploitation of glucose as an energy source, we assessed whether testosterone activates AMPK and glycolysis to increase glucose metabolism in cardiomyocytes. We found that testosterone promotes AMPK phosphorylation at Thr172, which is associated with increased protein activity [59, 60]. AMPK promotes glycolysis through increased glucose uptake by GLUT4 and enzyme activation (e.g., PFK2) [61]. Furthermore, AMPK delivers metabolic information through transcription factors for regulating gene expression of energy-encoded components that participate in ATP generation [60]. Metabolic transcription factors obtain metabolic information from primary energy sensors, such as AMPK, to coordinate energy demands and nutrient requirements with metabolic and growth-related gene expression programs [62, 63]. Furthermore, mitochondrial biogenesis is activated in response to changes in the AMP/ATP ratio and subsequent AMPK activation [42, 63].

Hypertrophic effects of testosterone require a series of complex metabolic changes involving activation of anabolic (energy consuming) and catabolic (energy producing) pathways. Thus, integration of both transcriptional and non-transcriptional mechanisms modulates complementary pathways in cardiac metabolism to meet the energy demand for cardiomyocyte growth. AMPK activity is variable and can be fine-tuned in response to different metabolic conditions. From this point of view, AMPK also is a mediator of the transcriptional outputs triggered by metabolic sensors, suggesting that these sensors, together with nuclear transcription factors, such as PGC-1α, might form a network controlling cellular energy expenditure [31, 42, 60]. AMPK upregulates GLUT4 expression via histone deacetylase (HDAC)5, and MEF2 appears to be the essential regulator of GLUT4 expression [64]. We and others previously showed that testosterone activates MEF2 in cardiomyocytes [41, 65]. Additionally, it has been shown that activation of AMPK by 5-aminoimidazole-4-carboxamide-1-beta-4-ribofuranoside (AICAR) blocks cardiac hypertrophy induced by several pro-hypertrophic stimuli, mainly through its inhibitory effect on the mTORC1 pathway [66, 67]. Interestingly, anabolic actions of testosterone involve mTORC1 activation in cardiac and skeletal muscle cells [54, 59, 68]. Thus, we assessed whether metabolic actions of testosterone are mediated by AMPK during the shift from normal to hypertrophied cardiomyocytes. Here, we determined that AMPK inhibition blocked testosterone-mediated glycolysis and hypertrophy. Similar results have been reported in prostate cancer cells, in which androgen treatment promoted cell growth, depending on AMPK [34]. Several studies have found that pharmacological and genetic activation of AMPKα inhibits cardiac hypertrophy in response to angiotensin II [69] and phenylephrine [70]. Antihypertrophic effects were associated with well-documented homeostatic roles of AMPK involving inhibition in protein synthesis by interfering with the mTOR pathway. In fact, it has been suggested that AMPK plays dual roles in the development of cardiac hypertrophy [71, 72]. In this context, submaximal AMPK activation or prior to hypertrophic stimulation may be antihypertrophic, whereas activation of AMPK during sustained and consolidated cardiac hypertrophy is necessary to maintain energetic metabolism and may be considered pro-hypertrophic [73]. Moreover, it has been suggested that AMPK activation prevents cardiac hypertrophy independent of mTOR by inhibiting microtubule accumulation [70] or by O-GlcNAcylation of structural proteins [71]. Conversely, adrenergic agonists increase AMPK activity in cardiac hypertrophy [74] and increased phosphorylation of AMPK at Thr172 is associated with anti-hypertrophic effects [73].

Moreover, it has been reported that hypertrophied hearts show activation of AMPK, which could be associated with energy requirements and dynamic metabolic adjustments in the hypertrophied heart [21]. Our finding for long-term testosterone exposure showed phosphorylation peaks at 3, 6 and 48 h. This result agrees with a potential oscillatory activity of AMPK responding to energy deprivation inside the cell [75]. It has been reported that pharmacological activation of AMPK with MK-8722 improves glucose homeostasis and induces cardiac hypertrophy [72]. As discussed above, to consider cardiomyocyte hypertrophy as an adaptive response, intracellular metabolism must be balanced during cell growth. Thus, AMPK-regulated energy metabolism during cardiomyocyte growth implies that anabolic environments are associated with controlled catabolic routes such as adjustments in glucose uptake and glycolysis [21].

In this study, we also determine whether glucose metabolism is regulated by androgens in an animal model of cardiac hypertrophy induced by testosterone. Accordingly, HK and PFK are targets of AR signaling, and these glycolytic enzymes increase in response to testosterone supplementation in ORX rats. HK2 is a glucose rate-limiting step enzyme and PFK2 is a primary regulator of cardiac glycolysis and substrate selection [19, 57]. PFK2 is activated through AMPK by phosphorylation, and this activity causes an increase in fructose-2,6-bisphosphate, which stimulates PFK1, further enhancing glycolytic pathway activity leading to increased glucose uptake and glycolysis [19, 31]. Therefore, transcriptional metabolic control mediated by AMPK-AR signaling appears to be a cellular mechanism that coordinates energy requirements with the testosterone-regulated growth machinery in cardiomyocytes.

Metabolic syndrome correlates with low testosterone plasma levels, suggesting a link between the anabolic effects of this hormone and heart failure associated to this metabolic disorder [76]. Clinical trials have depicted that administration of testosterone at physiological concentrations improved insulin sensitivity, central obesity, and heart failure progression in men suffering from metabolic disorders [2, 77]. Testosterone is also necessary to maintain normal insulin and glucose concentrations in the blood. Previous studies have shown a significant role for testosterone in the mobilization of GLUT4 to the plasma membrane in skeletal muscle, liver, and fat tissue [1, 78]. On the other hand, it has been reported that AR directly modulates the expression of both glycolytic and lipid-metabolic genes [79]. In transgenic testicular feminized mice, it was shown that lack of AR protein resulted in low mRNA expression of glycolytic genes (*Hk*, *Pfk*, and *Glut4*) in skeletal muscle, adipose tissue and liver, suggesting that AR plays a critical role on the modulation of glycolytic genes in these tissues [50]. Moreover, several new studies on the effects of physiological testosterone concentrations, which has been shown to produce beneficial effects and may become a treatments option for metabolic syndrome and heart failure as well as in aged-related cardiometabolic disorders. Because physiological actions induced by testosterone require changes of intracellular building-up machinery, protein synthesis and metabolic adjustments, these actions are considered primary events, which can be altered by the actions of DHT or estrogens in men, the effects of these androgen metabolites on glucose metabolism require further studies.

The results of this study show that testosterone influences the metabolic network played by AMPK-AR in cardiomyocyte hypertrophy. In addition, we determined that in testosterone-induced cardiomyocyte hypertrophy, glucose metabolism is enhanced and is regulated through a catabolic signaling pathway regulated by AMPK and anabolic signaling mediated by the AR. Studies aimed at establishing how energy-related signals are decoded by metabolic network dependent transcription factors that control cardiomyocyte growth will expand our understanding of the roles that anabolic hormones play in the cardiovascular system.

## Conclusion

A large number of studies in men over the past 30 years have shown that low circulating plasma testosterone levels correlate significantly with higher incidence of obesity, metabolic syndrome, type 2 diabetes mellitus and cardiovascular disease. Furthermore, testosterone replacement therapies improve these metabolic risk factors. In this study, we determine that changes in glucose metabolism triggered by testosterone during cardiomyocyte hypertrophy involve AMPK and AR pathway. Studies targeted to establish such effects at cellular level and their correlation with in vivo models will broaden our understanding of the roles played by anabolic hormones on the cardiovascular system. Thus, if testosterone influences glucose metabolism at a whole organism level, this could represent novel research approaches to study insulin resistance, obesity, diabetes and cardiovascular diseases.

## Supplementary Information

**Additional file 1: Figure S1.** High testosterone administration induces cardiac hypertrophy in vivo. ORX rats were treated with testosterone (ORX+T) or vehicle (ORX) Normal rats were used as control group. Cardiac hypertrophy was evaluated by heart weight/body weight ratio. Data are presented as means ± SEM. (n=4). *P < 0.05 vs. control; #P < 0.05 vs ORX group.

## Data Availability

The data used and/or analyzed during the present study are available from the corresponding author on reasonable request.

## Abbreviations

AMPK: AMP-activated protein kinase
AR: Androgen receptor
*β-mhc*: β-Myosin heavy chain
HK: Hexokinase
PFK: Phosphofructokinase
ECAR: Extracellular acidification rate
BrdU: 5-Bromo-2-deoxyuridine
CC: Compound C
Bica: Bicalutamide
Testo: Testosterone
ORX: Orchiectomy
CAMKII: Ca^2^^+^/calmodulin-dependent protein kinase II
GLUT4: Glucose transporter type 4
