# Supplementary figures and images for: Testosterone activates glucose metabolism through AMPK and androgen signaling in cardiomyocyte hypertrophy
Source: Biol Res. 2021 Feb 5;54:3. doi: 10.1186/s40659-021-00328-4 (PMC7863443; doi:10.1186/s40659-021-00328-4)

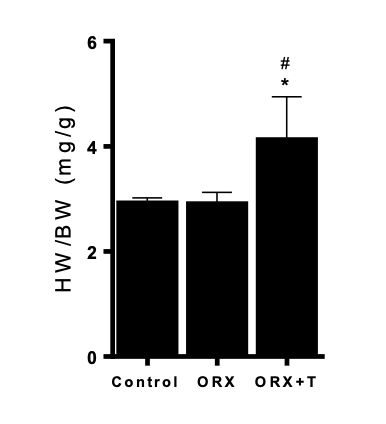

Supplement: Supplementary file 1 — Additional file 1: Figure S1. High testosterone administration induces cardiac hypertrophy in vivo. ORX rats were treated with testosterone (ORX+T) or vehicle (ORX) Normal rats were used as control group. Cardiac hypertrophy was evaluated by heart weight/body weight ratio. Data are presented as means ± SEM. (n=4). *P < 0.05 vs. control; #P < 0.05 vs ORX group. [file 40659_2021_328_MOESM1_ESM.png]
